# Stigma on Mental Health among High School Students: Validation of the Italian Version of the Attribution Questionnaire-27 (AQ-27-I) in a High School Student Population

**DOI:** 10.3390/ijerph17145207

**Published:** 2020-07-19

**Authors:** Silvia Ferrari, Cinzia Bressi, Elisa Busnelli, Giorgio Mattei, Sara Pozzoli, Anna Oliva, Gian Maria Galeazzi, Luca Pingani

**Affiliations:** 1 Department of Biomedical, Metabolic Sciences and Neurosciences, University of Modena and Reggio Emilia, Via del Pozzo 71, 41124 Modena, Italy; silvia.ferrari@unimore.it (S.F.); giorgio.mattei@unimore.it (G.M.); luca.pingani@unimore.it (L.P.); 2 Department of Neurosciences and Mental Health, Fondazione IRCCS Ca’ Granda Ospedale Maggiore Policlinico, Via Francesco Sforza 35, 20122 Milano, Italy; cinzia.bressi@unimi.it (C.B.); elisa_busnelli@hotmail.it (E.B.); sara.pozzoli@policlinico.mi.it (S.P.); oliva.anna84@gmail.com (A.O.); 3 Department of Economics “Marco Biagi”, University of Modena and Reggio Emilia, Via Jacopo Berengario 51, 41121 Modena, Italy; 4 Department of Health Professions, Azienda USL–IRCCS di Reggio Emilia, Via Amendola 2, 42122 Reggio Emilia, Italy

**Keywords:** psychometrics, stigma, social discrimination, high school students, mental health, stereotype

## Abstract

The purpose of this study was to describe the psychometric characteristics of the AQ-27-I in a high school student population. Students aged between 17 and 20 years and attending the fourth and fifth year of a scientific high school in Milan were approached at the school and were asked to fill in an anonymous socio-demographic form and the AQ-27-I. Cronbach’s alpha was used to estimate the instrument reliability and confirmatory factor analysis (CFA) was conducted and compared to the original English version factor structure. The AQ-27-I demonstrated acceptable internal consistency, with a Cronbach’s alpha of 0.87 and only one subscale (Personal responsibility) with an alpha lower than 0.60. Fit indices were very positive for the Dangerousness Model supporting the factor structure and paths of the original version. The Personal Responsibility Model, on the other hand, showed some weakness, concerning the process dynamics of the model. The results obtained are similar with those from other studies carried out in Italy and other countries. The questionnaire can be used for the quantitative description of stereotypes, emotions and behaviors associated with stigma in mental health in high school student populations.

## 1. Introduction

According to Goffman’s social theory, stigma is defined as a mark that reduces an individual “from a whole and usual person to a tainted, discounted one” [1]. Crocker et al. describe stigmatization as a phenomenon consisting in a negative connotation associated to a person or group that is supposed to possess “some attribute or characteristic that conveys a social identity that is devalued in a particular social context” [2]. Many people with mental disorders, including schizophrenia [3,4], experience stigma resulting from others’ lack of knowledge (stereotypes, ignorance or misinformation), attitudes (prejudice), or behavior (discrimination) [5,6,7,8,9]. The experience of stigma can lead to social exclusion [10,11,12,13], a decrease in self-esteem, due to the internalization of stigma [7,13,14,15,16], and the worsening of psychopathology, e.g., suicidal behavior [17]. Clear effects on the course and outcome of mental illnesses have been documented [7,9]: dealing with stigma is not only a humanitarian and ethical matter, but also a well defined and mandatory clinical action.

In recent decades, empirical research on the topic of stigma about mental disorders has significantly increased and different conceptual models have been developed [5,7]. Among these, public stigma (PS) was proposed by Corrigan and Watson [5]: PS was conceived as “the reaction that the general population has to people with mental illness”, thus distinguished from self-stigma, that is the stigmatizing attitude which people with mental disorder turn against themselves [18]. The model of PS is based on Weiner’s attribution theory [19,20], according to which stereotypes lead to assumptions regarding the personal responsibility of a person for their mental illness, which directly impacts on one’s emotions. If a person is perceived as responsible for their mental illness, then people may be angry at the person (for example, because it squanders public money for cures or treatments), and will marginalize them through segregation and coercion. If the person is perceived as not responsible for their condition, then feelings of pity will emerge. Another conceptual determinant of PS is the “Dangerousness Model”: if a person is considered dangerous because of their mental illness, then the general population is more likely to react with fears and to avoid them [21,22].

The Attribution Questionnaire 27 (AQ-27) was developed to measure PS through the model of “Personal Responsibility” and the “Dangerousness Model” [18] and has been validated in different languages [23,24] including Italian (AQ-27-I) [25], demonstrating good psychometric qualities.

Since adolescence is a critical period for the onset of mental illness [26,27,28], young people are important targets of mental health programs to overcome stigma. More positive attitudes toward mental illness and help seeking may lead to the earlier detection and delivery of treatment, and the significant improvement of outcomes [29,30,31]. Therefore, studies have recently focused on stigma among student populations [32]. The attitudes and beliefs of students about serious mental disorders seem not to substantially diverge from those of the general population. Dangerousness, unpredictability and incurability are the most common beliefs, and tend to be higher when schizophrenia is concerned, even among university students attending health-related professional training [33,34,35,36,37]. Students complain they do not have enough information about these conditions [34]. Furthermore, medical students believe that people with schizophrenia have no insight and cannot have a job or make sensible decisions regarding their lives [38].

Pilot studies or protocols intended to develop sustainable and effective mental health programs addressed to young people were developed [39,40,41]. A Californian study attested that teens tended to view blame and dangerousness as important variables leading to discrimination [42]. According to a study among Italian high school students [43], about 40% of the sample thought that a large fraction of people with mental illness can be dangerous; similar skeptical attitudes were recorded on the queries concerning the effectiveness of treatments and the likelihood of full recovery. Male and female students did not differ on the global measure of stigmatizing attitudes, yet female students were found to be more willing to help someone with a mental disorder and a trend towards more positive attitudes was observed in those with a family history of psychosis. Moreover, a better knowledge of mental illness was associated with higher willingness to provide help and with fewer stigmatizing attitudes.

The aim of this study was to describe the psychometric characteristics of the AQ-27-I in a student population of the last two years of a scientific high school in Milan. Although other Italian-language questionnaires useful for the quantitative evaluation of the stigma phenomenon in the high school population exist [43], and we believe that being able to use the same tool to compare different populations could be extremely useful in studying the stigma process.

## 2. Materials and Methods

### 2.1. Sampling and Recruitment

Students aged between 17 and 20 years, and attending the fourth and fifth year of a high school in Milan, were approached at the school, at the end of teaching classes, during the school year 2015/2016. The students were participating in an awareness-raising course on the topic of stigma and mental health. The participants were asked to fill in an anonymous socio-demographic form and the AQ-27-I, in paper version. Only fully completed questionnaires were considered in the study.

### 2.2. Measures

The AQ-27-I is a 27-item self-administered questionnaire, already validated in several languages (e.g., Italian, Spanish, Turkish) for use among the general population [23,24,25] and university students [35].

It consists of 9 subscales, each assessing the following stigma-related constructs: responsibility, pity, anger, dangerousness, fear, help, coercion, segregation and avoidance. Respondents are asked to rate their level of agreement with each statement about “Harry”, a 30-year-old single man with schizophrenia (for example: “I would feel unsafe around Harry”; “I would be willing to talk to Harry about his problems”; “I would think that it was Harry’s own fault that he is in the present condition”), on a Likert scale from 1 (“not at all”) to 9 (“very much”): higher scores indicate greater stigmatization. Six items, in the Italian version, are reverse scored (7, 8, 16, 20, 21, 26).

### 2.3. Statistical Analysis

The sample size was calculated using the ratio of at least 10 respondents to 1 item [44]: the AQ-27-I is composed of 27 items and therefore a sample of not less than 270 respondents is required. For this reason, the questionnaire was administered to 300 students of the last two years of a scientific high school in the city of Milan.

The psychometric characteristics of AQ-27-I were analyzed through Cronbach’s alpha coefficients (using George and Mallery rating score: ≥ 0.9—Excellent, ≥ 0.8—Good, ≥ 0.7—Acceptable, ≥ 0.6—Questionable, ≥ 0.5—Poor, and ≤ 0.5—Unacceptable [45]).

To verify the overlap between the theoretical models subtended by the questionnaire (“Model of Personal Responsibility” and “Dangerousness Model”), confirmatory factor analysis was used, and specifically for the following parameters: χ2, comparative fit index (CFI> 0.90), goodness of fit index (GFI > 0.90) and root mean square error of approximation (RMSEA < 0.05).

### 2.4. Ethical Statement

The study was not submitted for approval by a research ethics committee because it did not involve cases nor patients: the questionnaires used were administered to general population and do not produce diagnosis nor allow the definition of psychopathological conditions. Detailed information on the study was given to each participant and also consent was asked for the processing of personal data.

## 3. Results

Two-hundred and seventy-seven students agreed to participate in the study (92.33%) and their socio-demographic characteristics are summarized in Table 1. The sample was made up mostly of female (N = 200; 72.20%) students and the mean age was 17.78 (SD: ±0.70). Moreover, 21.66% (N = 60) of the students had a job (part-time or occasional), while 10.11% (N = 28) had a relative who had suffered, or suffers from, a mental disorder.

The Cronbach Alpha value for the questionnaire was 0.87, suggesting a good internal consistency. At the analysis of the values for each subscale, the values were all above cut-off for enough quality (0.60) except for the subscale ‘Personal Responsibility’ (0.40). Table 2 displays such values.

**Figure 1:**
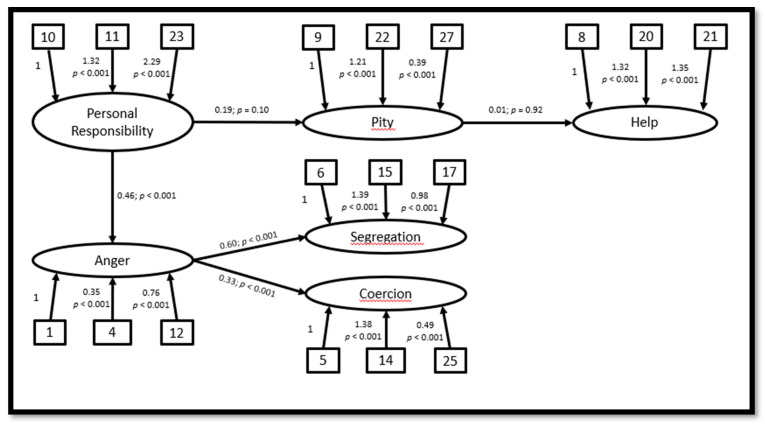
Factor loading analysis and the path analysis of the “Responsibility Model”.

**Figure 2:**
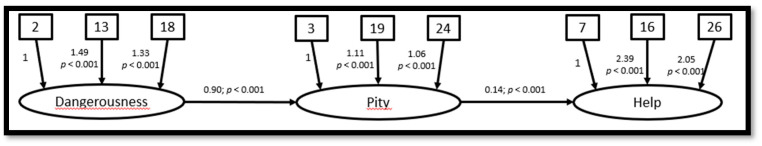
Factor loading analysis and the path analysis of the “Dangerousness Model”.

In Figure 1 and Figure 2 are described the factor loading analysis and pathway of the two theoretical models. The 27 items were positive, defined as loading in nine different first order latent factors: Personal Responsibility (10, 11, 23), Pity (9, 22, 27), Help (8, 20, 21), Anger (1, 4, 12), Coercion (5, 14, 25), Segregation (6, 15, 17), Dangerousness (2,13,18), Fear (3,19,24) and Avoidance (7,16,26). In the Responsibility Model, the regression weights are statistically significant except for “Personal Responsibility–Pity” and “Pity–Help”; for the Dangerousness Model, however, they are all statistically significant. Indices for the path model representing the theory of dangerousness are extremely encouraging: the chi-square test supported the fit (Χ^2^ = 0.28; df = 1; *p* = 0.60), CFI = 0.99, GFI = 0.99 and RMSEA = 0.001. On the other hand, the indices are not so positive for the personal Responsibility Model: the chi-square did not support the fit (Χ^2^ = 126.29; df = 10; *p* < 0.001), CFI = 0.44, GFI = 0.89 and RMSEA = 0.234.

## 4. Discussion

The aim of the present study was to validate the AQ-27-I in a population of a scientific high school: our results shown good psychometric properties and confirmed the AQ-27-I as a useful tool to quantify stigmatizing attitude in a high school population.

The Cronbach α values for AQ 27-I were acceptable, demonstrating a significative internal consistency: excellent for the subscale “Fear”, good for the subscale “Dangerousness” and for the total score total score. These results are encouraging as they are better than those obtained in a previous validation of the same questionnaire in the general Italian population [25], where three subscales reported an internal consistency of less than 0.60 (“Anger”, “Coercion” and “Avoidance”). Furthermore, Cronbach’s Alpha relating to the total score (0.87) was higher than it was for both previous validations in the Italian language: 0.81 for the general population [25] and 0.68 for a university student population [35].

The pathway analysis has shown very good strength for the Dangerousness Model confirming what has already been verified in the validation studies mentioned above: all the fit indices were abundantly above the required cut-offs, confirming its adaptability to the Italian context. The same model has obtained excellent results in Turkish [23] and Spanish [24] languages, where the fit indices and the regression weights have been decidedly above the normative values. The validation of the Responsibility Model was more problematic: although the regression weights indicated the same direction in the associations between the different subscales of the original English validation [18,46], the fit indices that describe the model were below the required values. However, these results are in continuity with the problems encountered in the validation in the general population in Italian but also in Turkish [23] and Spanish [24] languages: we can hypothesize that this model, solid in the English language and in the American setting, does not find a full correspondence in other cultural contexts. In our previous study [35] we showed how the same model, in the university student population, expressed better fit indices if adapted in three different sub-models: (a) Personal Responsibility → Pity → Help; (b) Personal Responsibility → Anger → Coercion; and (c) Personal Responsibility → Anger → Segregation.

Some limitations of the work here described must be acknowledged: firstly, the student sample was collected in a high school, usually attracting teenagers from higher socio-cultural backgrounds, that may not be representative of the whole population of the same age. Secondly, the sample was characterized by a high percentage of female students (N = 200; 72.20%) and this may have influenced our findings. For example, the high school female students were found to be more willing to help someone with a mental disorder [43]. Thirdly, the populations investigated come from a precise geographical location in the North of Italy, and therefore may not represent the entire Italian population. Finally, the process of the building up of stigma is very complex, as influenced by many variables (ethnic, cultural, religious, economic, etc.): these were not included as potential confounding variables. Therefore, moderate caution should be exerted when considering the results of the models.

## 5. Conclusions

This study tried to validate AQ-27-I in a specific context (high school students): the results obtained are very similar with other studies carried out in Italy and in other countries. The questionnaire can be used for the quantitative description of stereotypes, emotions and behaviors associated with stigma in mental health in high school students. Instead, more caution is needed using the questionnaire to define the stigma process: the model of dangerousness is strong while that of personal responsibility needs further study for a better definition. Though it is known that effective interventions to reduce stigma are very hard to develop [47], it may be that a more diffuse use of tools like AQ-27 within educative programs may contribute to increase awareness and positive thinking on these issues.

## Figures and Tables

**Table 1:** Socio-demographic characteristics of the sample.

	Mean; SD; Range
*Age*	17.78; ±0.70; 17–20
	N (%)
*Sex*	
Male Female	77 (27.80%) 200 (72.20%)
*Job*	
Paid work No paid work	60 (21.66%) 217 (78.34%)
*Positive psychiatric family history*	
Yes No	28 (10.11%) 249 (89.89%)

**Table 2:** Cronbach’s Alpha values.

	Cronbach’s Alpha
*Total Score AQ-27-I*	0.87
*Personal Responsibility*	0.40
*Pity*	0.71
*Help*	0.77
*Anger*	0.60
*Coercion*	0.61
*Segregation*	0.75
*Dangerousness*	0.81
*Fear*	0.91
*Avoidance*	0.64
